# L-shaped corticotomy with bone flap sliding in the management of chronic tibial osteomyelitis: surgical technique and clinical results

**DOI:** 10.1186/s13018-019-1086-0

**Published:** 2019-02-12

**Authors:** Teng-fei Lou, Gen Wen, Chun-yang Wang, Yi-min Chai, Pei Han, Xiao-fan Yin

**Affiliations:** 1 Orthopaedic Department, Shanghai Jiao Tong University Affiliated Sixth People’s Hospital, Shanghai, People’s Republic of China; 2 Orthopaedic Department, Minhang Branch, Zhongshan Hospital, Fudan University, Shanghai, People’s Republic of China

**Keywords:** Tibial osteomyelitis, L-shaped corticotomy, Bone flap, Bone transport

## Abstract

**Background:**

We described the use of the technique of L-shaped corticotomy with bone flap sliding to treat chronic osteomyelitis of the tibia in eight patients and presented the preliminary results.

**Methods:**

L-shaped corticotomy with bone flap sliding was performed in eight patients between 2007 and 2014. All patients had chronic tibial osteomyelitis involving the anterior tibial cortex with intact and healthy posterior cortex. The size of bone defects following sequestrectomy and radical debridement was 8.1 cm on average. One patient required a latissimus dorsi flap. The mean follow-up period was 34.1 months. The functional and bone results were evaluated at the time of the latest follow-up.

**Results:**

Complete eradication of infection and union of docking sites were achieved in all patients. Functional results were judged excellent in five patients and good in the rest three patients. Bone results were graded as excellent in all cases. The mean external fixation time was 169.9 days and external fixation index was 21.2 days/cm. Pain was the most common complaint that we faced during lengthening. Pin tract infections were observed in four patients, and mild transient stiffness of ankle joint was observed in three patients.

**Conclusions:**

We have found this technique to be safe and effective, significantly diminishing the external fixation index. The earlier removal of the external fixator may result in increased patient comfort, a reduced complication rate, and a rapid and convenient rehabilitation.

## Introduction

Chronic osteomyelitis of the tibia, which can be post-operative or secondary to open fracture [1], remains a considerable challenge to treat in clinical practice. Chronic osteomyelitis typically results in necrosis of soft tissues and bone to a variable extent. The necrotic bone forms infected foci for hosting pathogens. Furthermore, the host anti-infection mechanisms are frequently not in an ideal condition to resist microorganisms, and the poor circulation makes it hard to deliver an effective concentration of antibiotic to the infection site [2].

In order to create a vascular and viable environment, the first step of managing a chronic osteomyelitis is appropriate radical debridement requiring excision of all infected bone and soft tissue [3]. However, radical debridement and sequestrectomy often result in massive segmental bone loss and limb shortening. There are several different surgical techniques reported to treat chronic osteomyelitis, including antibiotic cement rod [4], vascularized bone graft [5], bone grafting [6], and Ilizarov methods [7].

In contrast to other methods, Ilizarov technique of distraction osteogenesis allows radical debridement and sequestrectomy and can solve not only the segmental defect but also the coexisting problems of deformity, limb shortening, joint contractures, and soft tissue loss [1, 7]. Therefore, Ilizarov bone transport has gradually became a main treatment for tibial chronic osteomyelitis.

Although Ilizarov technique is associated with satisfactory results in most cases, one of the most common problems of this method is the prolonged duration the external fixator has to be retained in place until the newly generated bone consolidates completely, which relates to many complications including infection of pin-tract, loosening of distractor device, stiffness of joint, persistent pain, refracture, angulation, and delayed union at the docking site [1, 7].

As is well-known, an intact vascular bed and a rich blood supply are conducive to curing chronic osteomyelitis [8]. The technique of L-shaped corticotomy with vascularized bone flap sliding can preserve blood supply from both the osteotomic and debridement area to the largest possible extent and increase the bone contact area, thus shortening the duration of Ilizarov distraction device and solving the problems caused by traditional method of bone transport. We will now describe in a stepwise manner our surgical technique and present the clinical results.

## Patients and methods

This was a retrospective study approved by the Ethics Committee of Shanghai Jiao Tong University Affiliated Sixth People’s Hospital. All procedures were in compliance with the Helsinki Declaration. Informed consent for participation was obtained from all participants in this study.

Between 2007 and 2014, eight consecutive patients with chronic osteomyelitis only involving the anterior tibial cortex were treated using the technique of L-shaped corticotomy with partial bone flap sliding at our hospital (Table 1). Diffuse osteomyelitis affecting both anterior and posterior cortices of the tibia is contraindications to this procedure. Severe neuro-vascular damage or mental disease or any other conditions which would bring about the lack of cooperation are also contraindications.

**Table 1:** Patient data

Patient no./sex/age(years)	Initial causes	Symptoms (months)	Site	Type of infection	Organism grown on culture	Operations before distraction device application	No. of previous operations	Comorbidities	Soft tissue defect at presentation	Bone defect (cm)	Nicotine abuse
1/M/33	Open tib.fract.	28	Tib, D	Active drain	*S. aureus*	EF→WD→WD→IF+BG→WD→SG→WD→WD	8	None	Yes	9	No
2/M/60	ORIF	15	Tib, D	Active drain	*S. aureus*	IF→WD→WD→IF+BG→WD	5	Hypertension	No	7	Yes
3/F/27	ORIF	6	Tib, P	Active drain	*E. coli*	IF→WD→WD→WD	4	None	No	5	No
4/M/42	Open tib.fract.	10	Tib, D	Active non-drain	*S. aureus*	EF→WD→IF+BG→WD→WD	5	None	No	9	Yes
5/M/25	ORIF	4.5	Tib, D	Active drain	*Pseudomonas*	IF→WD	2	None	No	7	No
6/M/40	ORIF	9	Tib, P	Active drain	*S. aureus*	IF→WD→WD+BG→WD→WD	5	None	No	8	Yes
7/F/30	ORIF	23	Tib, S	Active non-drain	*Pseudomonas*	EF→IF+BG→WD→WD→MFCF→WD	6	None	No	11	No
8/M/38	Open tib.fract.	120	Tib, D	Active drain	MRSA	EF→WD→WD→IF+BG→WD→WD→MFCF→WD→WD→WD	10	None	Yes	9	No

*ORIF* open reduction and internal fixation, *Tib* tibia, *D* distal, *P* proximal, *S* shaft, *MRSA* methicillin-resistant *Staphylococcus aureus*, *EF* external fixation, *WD* wound debridement, *IF* internal fixation, *BG* bone grafting, *SG* skin grafting, *MFCF* musculofasciocutaneous flap

An average of 5.6 operations was performed in these cases prior to application of an Ilizarov fixator. Two patients (cases 1 and 8) had combined soft tissue defects (4 cm × 5 cm in case 1 and 3 cm × 5 cm in case 8) which we treated with open dressing changing; we hoped that the healthy skin would transfer simultaneously with the bone flap to cover the wounds.

### Surgical technique

#### Stage I (preoperative preparation, hardware removal, and radical debridement)

Deformity, shortening, distal neurovascular status, local skin condition, joint function, and nutritional index are evaluated. Laboratory examinations including CRP (C-reactive protein), ESR (erythrocyte sedimentation rate), and WBCC (white blood cell count) are taken to measure active infection.

**Fig. 1:**
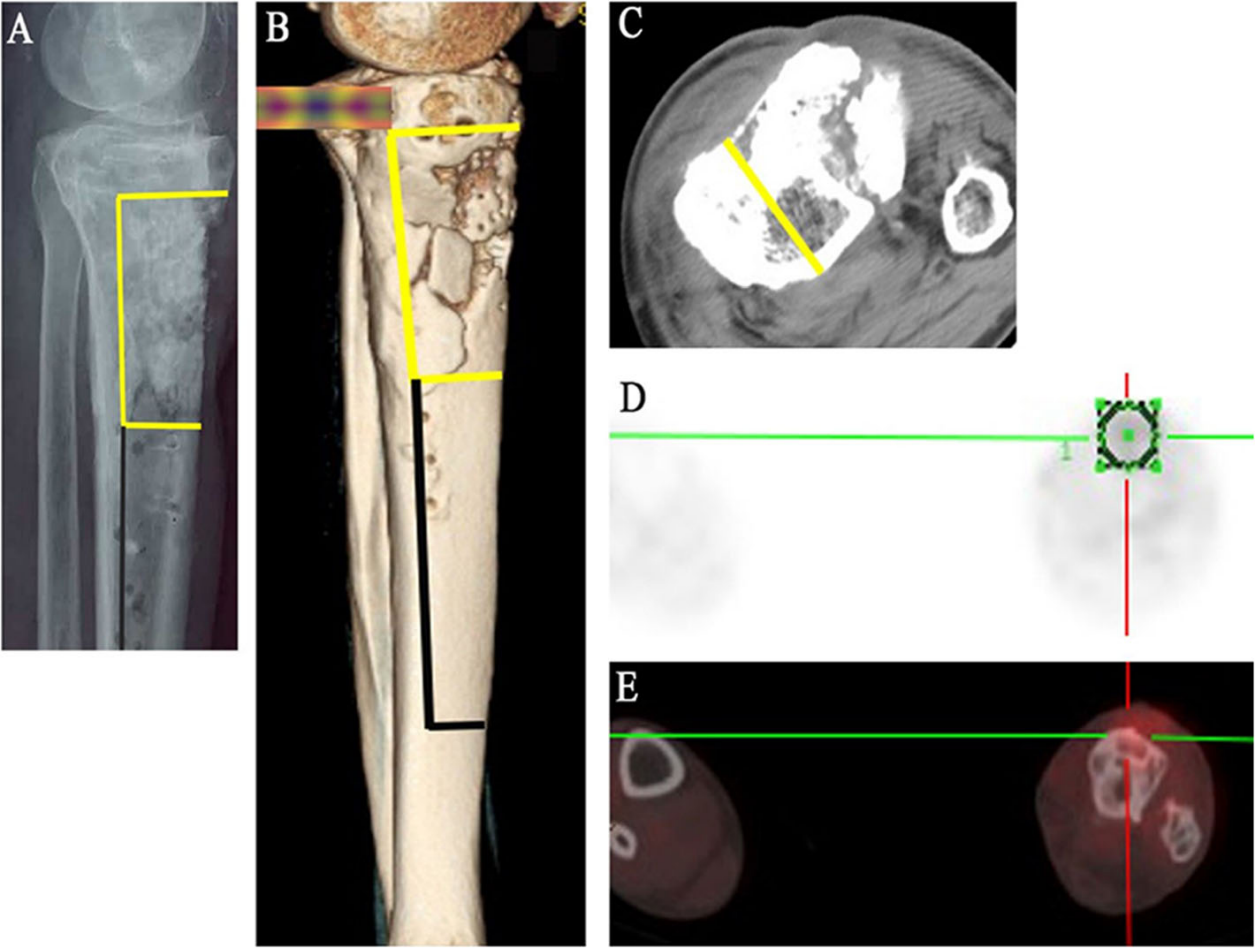
**a**–**g** A 60-year-old man with chronic osteomyelitis of the left tibia. Imaging examinations are taken to assist in determining the extension of infection and resection. **a** A preoperative lateral radiograph shows the extension of the infection that compromises the proximal diaphysis and margins of resection (yellow line) and corticotomy (black line) is determined. **b**, **c** The CT scans show the extension of bone destruction and margins of resection (yellow line) and corticotomy (black line) is determined on lateral (3D) and coronal images. **d**, **e** The PET-CT scan shows increased uptake in the area of infection

Direct radiographs, CT (computed tomography), MRI (magnetic resonance imaging), and PET-CT (positron emission tomography-computed tomography) are performed in the preoperative assessment of the probable extent of infection or dead bone (Fig. 1a–e). All these imaging examinations help to ensure that the infections only involve the anterior tibial cortex and determine the resection levels. However, if the appearance of the bone is found unsatisfactory during intraoperative observation, more extensive resection is performed than it has been planned based on the preoperative studies, and conventional technique of total segment resection is used when necessary.

**Fig. 2:**
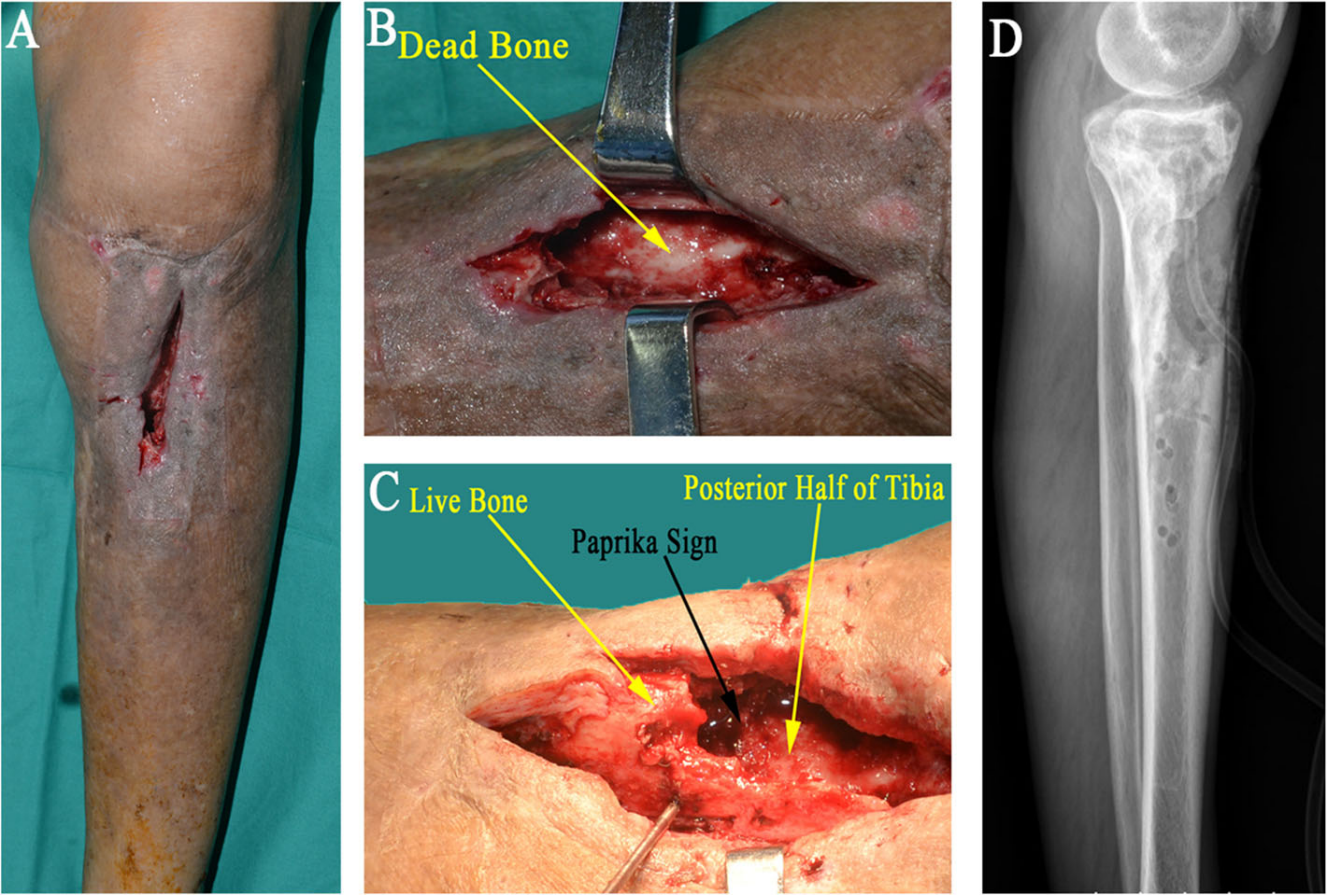
Debridement technique. **a** Clinical photograph of the affected leg. **b**, **c** During debridement, the infected or necrotic bone is removed radically until the “paprika sign” (cortical bone bleeding area) appears. It is of great importance to make sure that the posterior half of tibia retained following the radical debridement is uninfected and intact. **d** Lateral radiograph shows bone defect following resection of dead bone

The patient is maintained in supine position on a radiolucent operating table (Fig. 2a). The incision is made containing the sinus tracts. The hardware is removed in case where plate, screws, or intramedullary nail from previous surgery exist. Sinus track for all necrotic bone and soft tissue are obtained for tissue cultures. Then, the infected or necrotic bone is removed radically until the “paprika sign” (cortical bone bleeding area) appears (Fig. 2b and c) [9]. The infected fibrotic scarred tissue surrounding the infected bone is also excised. It is of great importance to make sure that the posterior cortex retained following the radical debridement is uninfected and intact. In order to preserve as much vascular bed as possible, periosteal stripping is only confined to the area determined to be resected; thus, the posterior and lateral tibia periosteal attachment where the soft tissue will provide considerable vascularization is kept intact. Hydrogen peroxide, iodine liquid, and saline are used one after another to flush the wound resulted from debridement and sequestrectomy, after which iodine liquid immersion is carried out for 10 min, and then, saline irrigation is implemented again to make the wound clean. The incision is closed with drainage tubes. If the infected area has large soft tissue defect, vacuum sealing drainage (VSD) or open dressing changing is made to close the wound.

Antibiotics that are suitable according to the results of cultures and antibiotic susceptibility tests are applied intravenously for at least 3 weeks or until the ESR and CRP levels return to normal limits.

#### Stage II (application of external fixator and L-shaped osteotomy)

Before the second stage, the infection is determined completely eradiated through three aspects. Firstly, the wound is inspected and it is clean without any sign of infection such as increased pain, redness, and purulent appearance secretion within 3 to 6 weeks. Secondly, normal ESR and CRP levels are attained. Thirdly, biopsy specimens obtained from the gap left after debridement as a percutaneous procedure are sent for frozen-section and Gram-staining analysis. Total resolution of infection is demonstrated by < 5 polymorphonuclear leukocytes per high-power field and the absence of microorganisms on Gram-staining [9].

**Fig. 3:**
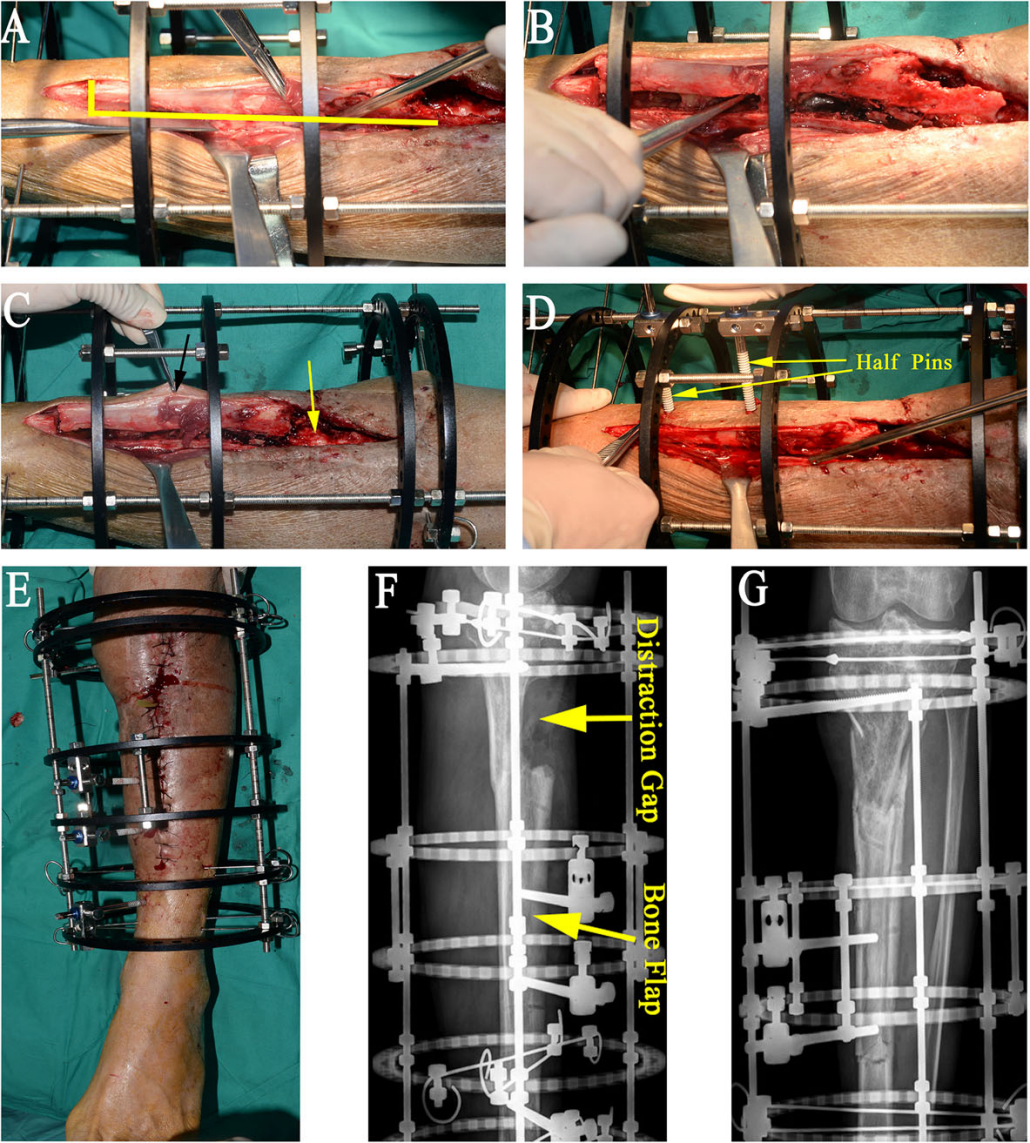
Application of external fixator and L-shaped osteotomy. **a** The holes drilled by Kirschner wires form an L-shaped configuration (yellow line), and osteotome is used to separate the bone flap. **b** The bone flap is separated from the posterior half of tibia. **c** The bone flap is not separated from the soft tissue it attaches to, thus being provided with good blood supply from the anterior and lateral soft tissue (black arrow), and remaining posterior cortex after debridement and sequestrectomy keeps the periosteal attachment intact and also ensures a good blood supply (yellow arrow). **d** Two 5.0-mm half pins are used for unilateral cortical fixation of the bone flap. **e** The incision is closed with drainage tube. **f**, **g** Immediate postoperative latera and anteroposterior radiographs

The L-shaped corticotomy starts with a longitudinal incision made over the anteromedial surface of the tibia. After the tibial shaft is exposed, Kirschner wires are used to drill holes, the connection of which is parallel to tibia posterior border and keeps in alignment with the front edge of the posterior cortex retained in the debridement area. All the holes form an L-shaped configuration along which an osteotome is used to separate the bone segment which will be transferred (Fig. 3a, b). The bone segment created for transfer is not separated from the soft tissue it attaches to (Fig. 3c), thus being provided with good blood supply from the anterior and lateral soft tissue, so we call it “vascularized bone flap.” The length of the bone flap created for sliding is about 12 cm. No specific criteria are used to determine exactly how long the bone flap should be. Two or three 5.0-mm half pins are used for unilateral cortical fixation of the bone flap (Fig. 3d). The incision is closed with drainage tubes (Fig. 3e). If the infected area has soft tissue defect which may well heal with the transport of the soft tissue surrounding the bone flap, open dressing changing is made to cover the wound.

**Fig. 4:**
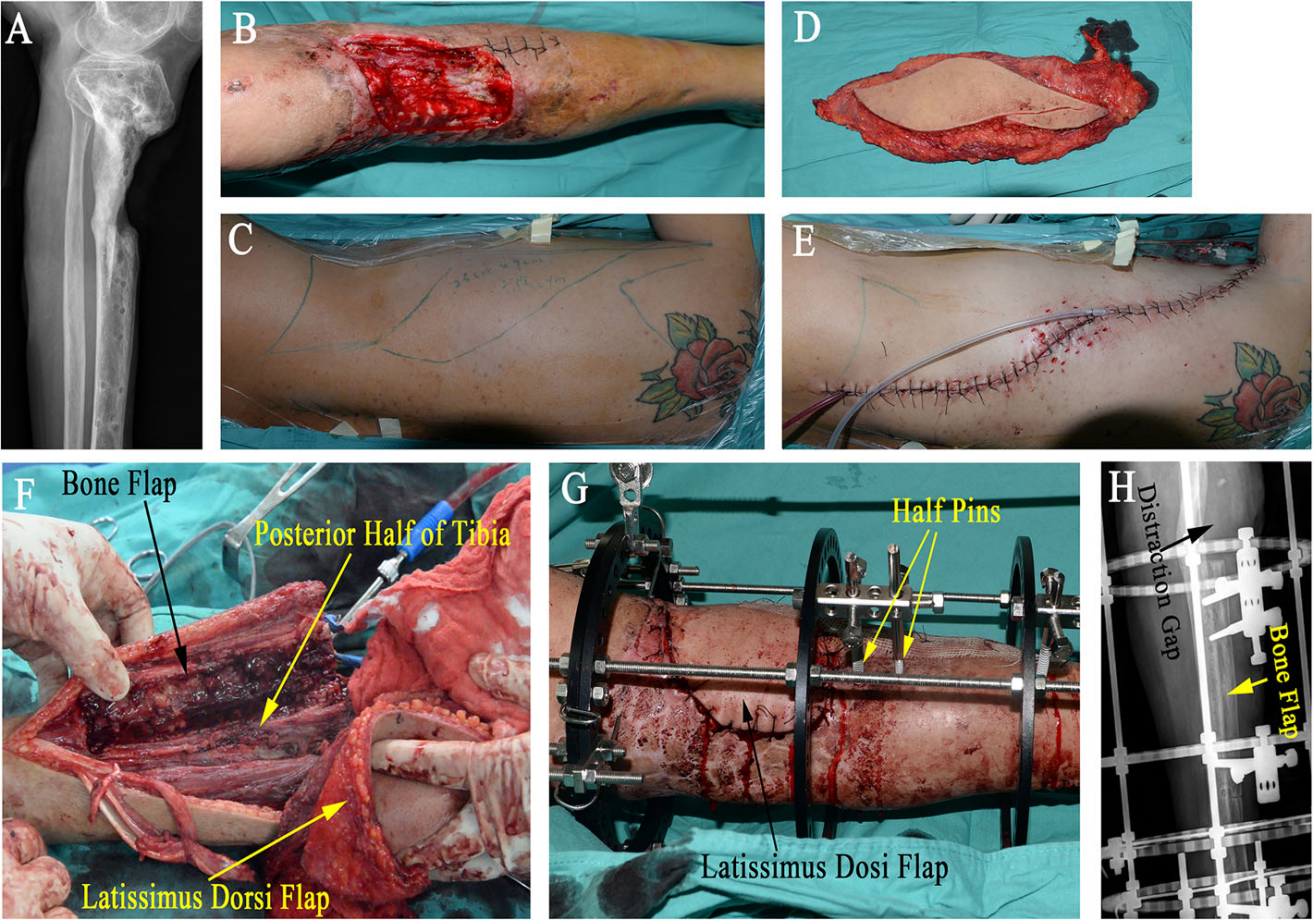
A 33-year-old man with chronic osteomyelitis of the left tibia. **a** Radiograph shows bone defect following resection of dead bone. **b** Soft tissue defect following debridement. **c** Planning on the harvest of free latissimus dorsi flap. **d** The latissimus dorsi flap is harvested. **e** The donor site is sutured directly drainage tubes. **f** The bone flap is separated from the posterior half of tibia, and one tip of the flap is sewed up with the wound edge. **g** The latissimus dorsi flap completely covers the soft tissue defect, the external fixator is assembled, and two half pins are used to fix the bone flap. **h** Immediate postoperative lateral radiograph shows distraction gap and bone flap

**Fig. 5:**
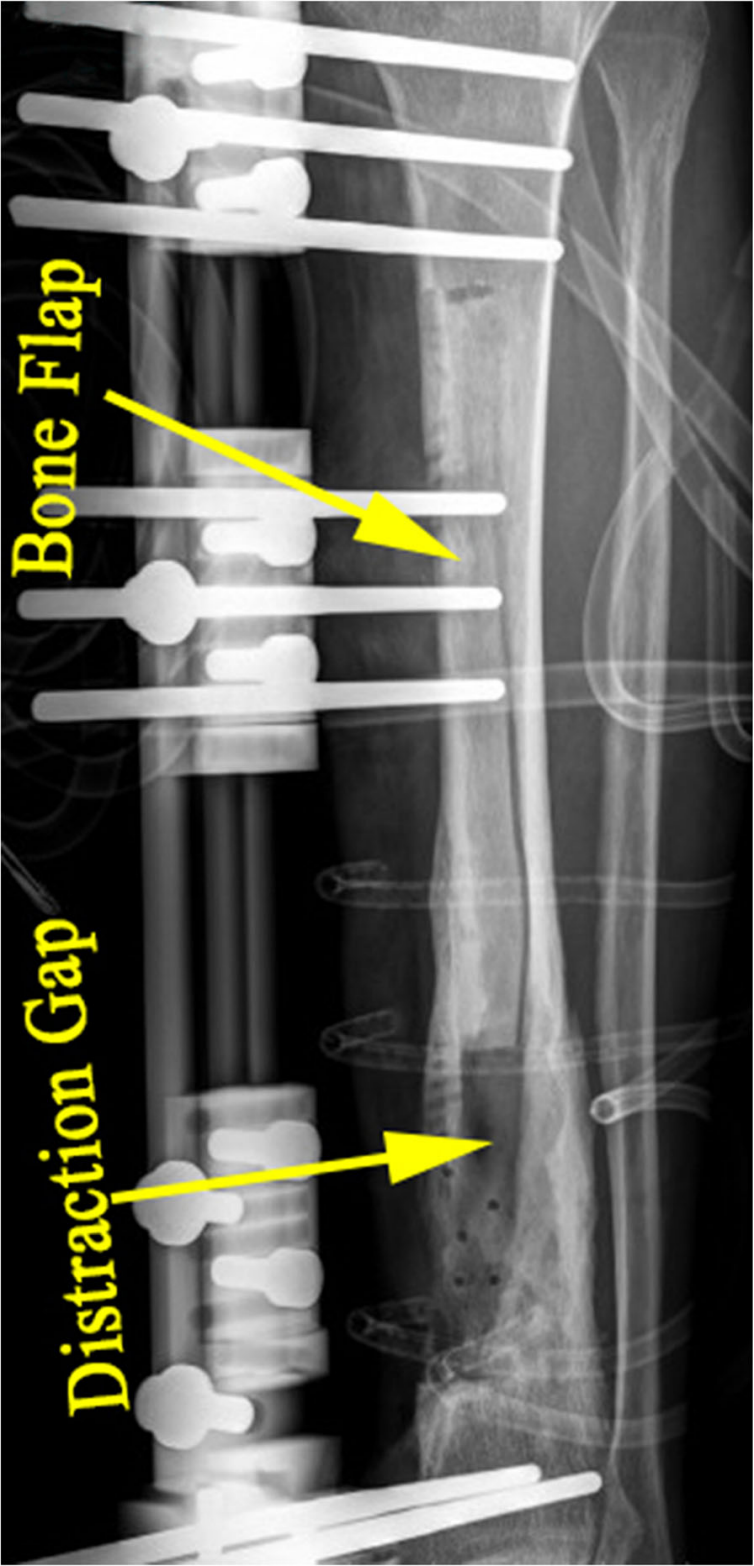
Postoperative lateral radiograph of the patient treated with monolateral external fixator

The external fixator is applied either before or after corticotomy. Empirically, if there is no soft tissue defect in the infected area or the defect is small, external fixator is applied before the L-shaped corticotomy. Otherwise, if there is existing large soft tissue defect which requires flap to cover, external fixator is normally applied after corticotomy and flap grafting in order to make it easy for flap design and suture (Fig. 4a–h). Two kinds of external fixators are applied depending on the conditions of the fibula of the affected leg. Normally, if the fibula of the affected leg is intact, either Ilizarov ring fixator or monolateral external fixator (Fig. 5) is used; otherwise, Ilizarov ring fixator is applied for fear that the remaining posterior cortex is not stable enough without fibular to divert the pressure.

#### Stage III (postoperative care and removal of the external fixation)

Distraction of 1.0–1.5 mm per day is begun after a latency period of 3 to 5 days. The rhythm of distraction is determined by pain reaction and the quality of newly generated bone between distraction gap evaluated according to the first few radiologic examinations. Since docking is completed, the compression between docked ends is kept on with the rhythm of 0.5 mm per day for 10 days in order to get full contact. Gentle range of motion exercises of knee-joint and ankle-joint is encouraged on the second day after surgery. Gradual partial weight-bearing is performed during treatment, and active full weight-bearing is allowed when docking is achieved. Intravenous sensitive antibiotic is given postoperatively for 1 to 2 weeks. The monolateral or Ilizarov external fixator is removed when solid union of docking site is showed by radiographs.

### Postoperative follow-up

Clinical follow-up was performed every 2 weeks to check pin tract condition, frame stability, and range of motion of adjacent joints. Radiographs were carried out every 2 weeks during the distraction phase and monthly during the consolidation phase for the assessment of bone union and quality of consolidation. Laboratory examinations including ESR, CRP, and CBC values were evaluated at appropriate times to ensure eradication of infection. Postoperative complications were recorded.

The assessment of pin tract inflammation was made according to Dahl’s grading [10]. Evaluation of bone results and functional results was made according to the criteria of Paley et al. [11]. The external fixation time (EFT) represented the total number of days the external fixator was attached to the bone. The external fixation index (EFI) was defined as the duration of external fixation in days divided by the total amount of lengthening in centimeters.

## Results

**Fig. 6:**
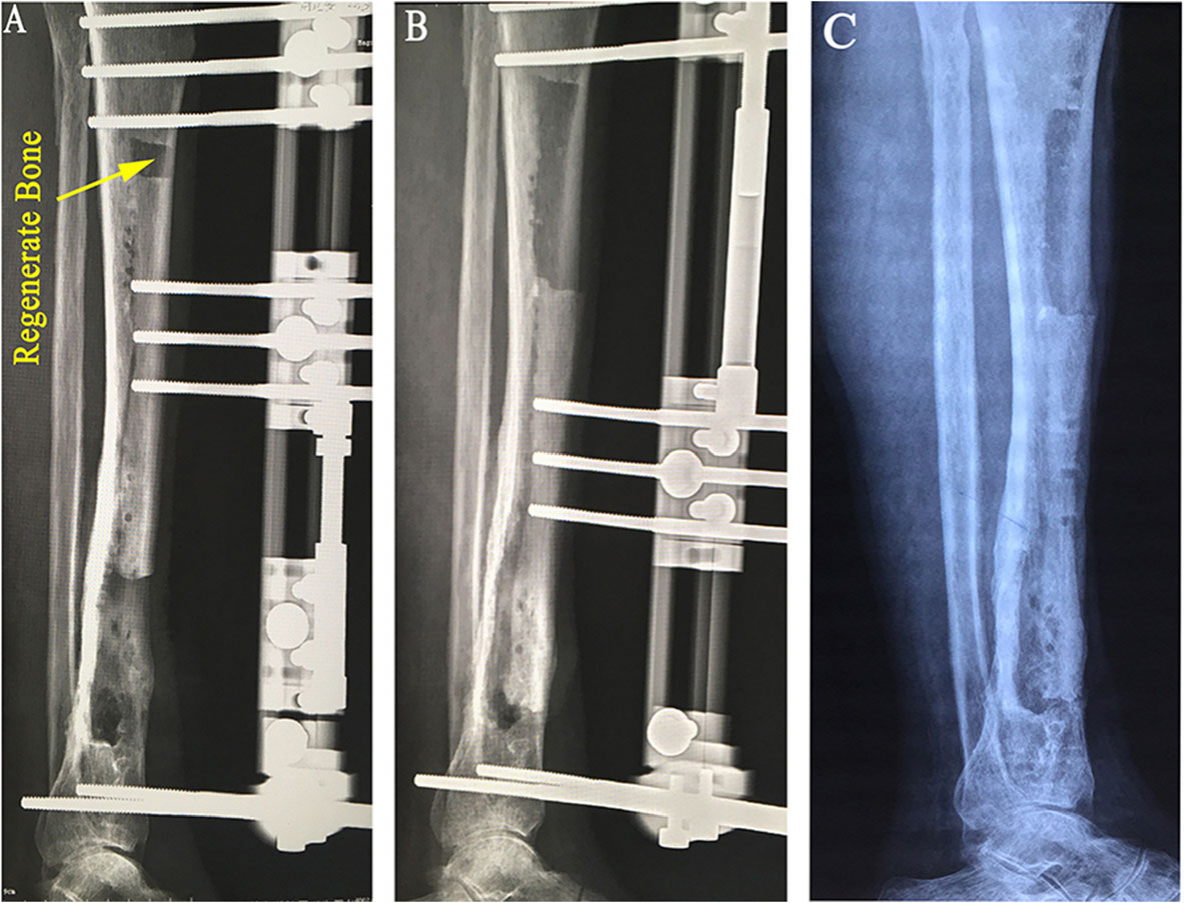
**a** Twenty-five days after operation with partial bone flap sliding. **b** Ninety days after operation, bone ends had contacted with each other and the regenerate bone had commenced to be mineralized. **c** One hundred eighty-five days after operation, consolidation of the newly formed bone and union of the docking site were presented, and the fixator was removed

Complete eradication of infection and union of docking sites were achieved in all patients. A mean lengthening of 8.1 cm was achieved at an average follow-up of 34.1 months (23–51). The mean EFT was 169.9 days (112–215) and EFI was 21.2 days/cm (16–27.6). Based on the criteria recommended by ASAMI, functional results were judged excellent in five patients and good in the rest three patients. Bone results were graded as excellent in all cases (Table 2 and Fig. 6a–c).

**Table 2:** Results at latest follow-up

Case no.	Duration of follow-up (months)	Lengthening achieved (cm)	Union of docking site	Complications	Recurrence	Functional results	Bone results	EFT (days)	EFI (days/cm)	Additional surgical procedures
1	32	9	Yes	Pain, PTI, AJS	No	Good	Excellent	185	20.6	No
2	23	7	Yes	Pain	No	Excellent	Excellent	112	16	No
3	51	5	Yes	Pain	No	Excellent	Excellent	138	27.6	No
4	29	9	Yes	Pain	No	Good	Excellent	210	23.3	No
5	31	7	Yes	Pain, PTI	No	Excellent	Excellent	153	21.9	No
6	42	8	Yes	Pain	No	Excellent	Excellent	177	22.1	No
7	39	11	Yes	Pain, PTI, AJS	No	Good	Excellent	215	19.5	No
8	26	9	Yes	Pain, PTI, AJS	No	Excellent	Excellent	169	18.8	No

*PTI* pin tract infection, *AJS* adjacent joint stiffness

Pain was the most common complaint that we faced during lengthening, and it was quite severe for the first few days in some patients after operation. Almost all patients had a feeling of dull pain during the distraction period, especially during walking or at night. Most of them were relieved of pain by oral analgesics. One patient who could not tolerate the pain was treated with decreasing the rate of lengthening. Pin tract infections graded II and III according to Dahl’s classification were observed in three patients. These superficial infections were resolved with local pin care and oral administration of broad-spectrum antibiotics. Grade IV pin traction infection was detected in one patient who had a purulent discharge and finally controlled by intravenous sensitive antibiotic according to bacteria culture with pus. Uncontrollable infection that required fixator extraction was not observed. Though three patients developed mild transient stiffness of ankle joint at the time of removal of the fixator, all of them achieved full range of motion in the ankle after an average of 2 months of physiotherapy.

There were two patients combining soft tissue defect that we expected to heal with simultaneous skin transfer; however, the defect remained when the docking was achieved, and only the wound area was downsized (1 cm × 2 cm in case1 and 1 × 1 cm in case 8). The tweezers did not reach the surface of tibia when we used them to explore the wounds. We decided to keep on changing dressing in consideration of that the wounds were clean without any sign of infection, the granulation tissue was fresh, and the results of bacterial cultures were both negative. Eventually, it respectively took 42 days and 28 days after the docking was achieved for the wounds to heal.

Intraoperative complications, angulation, and delayed consolidation of the docking site, joint contracture subluxation, or wire breakage did not occur in any of the patients during the treatment. No refracture, leg length discrepancy, and neurovascular damage were observed during follow-up.

## Discussion

The treatment of chronic tibial osteomyelitis is a thorny problem in orthopedic surgery. Patients determined to accept limb salvage treatment usually undergo multiple previous surgical interventions, causing soft tissue compromise and bone defects. In the last few years, Ilizarov technique of distraction osteogenesis has gradually became a main method to treat tibial chronic osteomyelitis. Comparing with other methods, Ilizarov bone transport could solve not only the segmental defect but also the coexisting problems. However, limb salvage option with conventional Ilizarov technique is time-consuming for both the surgeon and the patient and is associated with many complications due to the prolonged treatment time. The main aim of the present study was to investigate a modified technique to obtain elimination of infection and to substantiate that this technique can diminish the EFI and reduce the risk of complications.

It is hypothesized that the process of distraction osteogenesis could trigger a robust and significantly great vascular response which could potentiate healing of osteomyelitis or of distant hypovascular nonunions [12]. This hypothesis has been proved by the results of a study carried out by Donneys. He reported a 1.93-fold increase in vessel volume fraction and a 1.73-fold increase in vessel number within regenerate area existing at 40 days post-osteotomy in the process of bone lengthening compared to the process of normal fracture healing [13]. Thus, it can be seen that abundant blood supply makes a critical contribution to the healing of osteomyelitis during distraction osteogenesis. One advantage of our method of L-shaped corticotomy with vascularized bone flap sliding is that the blood supply from both the osteotomic and debridement area is preserved to the largest possible extent, which may promote the formation of new bone and improve the mineralization and density of the regenerate, thus accelerating the process of both distraction phase and consolidation phase during distraction osteogenesis. The results of our study showed that even five of our patients underwent distraction at a rate of 1.5 mm per day, the quality of bone formation was not negatively affected.

The docking site is prone to nonunion or delayed union when the contact area between the fragment ends is not large enough. There are several viable options recommended for preventing this common complication and reducing the duration of fixator. Some surgeons refreshed the bony edges with osteotome and curette and some performed autogenous bone grafting at the docking site [14, 15]. Transport over an intramedullary nail [16] and bifocal transport [17] also have been reported to be effective. However, our surgical technique provides a wilder contact area between the bone flap and normal bone compared with traditional technique, which may avoid nonunion or delayed union of the docking site. Therefore, bone edges refreshment, bone grafting, or other further operation may not be necessary.

Thanks to the posterior half of the tibia left during debridement, another great advantage of this technique in comparison to the conventional technique may be the ability of the patients to bear weight and to be ambulatory better and earlier during treatment, and thus, some related complications such as refracture of the regenerated bone, angulation at the docking site, and deformity, which are complications usually resulted from excessive functional exercise or overload of weight bearing, may be avoided. Also, discrepancy is unlikely to occur with our method because the length of tibia will remain the same during the whole course of treatment.

Given the above, rich blood supply, satisfactory union of the docking site, and bearing weight safely and early helped our technique achieve notably reduced EFI and satisfactory bone and functional results compared with conventional technique. Studies on the classic bone transport with Ilizarov external fixator in the management of nonunions associated with infection have reported an average EFI of 54.9 days/cm [9], which is significantly longer than the average EFT in the current study of 21.2 days/cm. Also, early dismantlement of the external fixator significantly reduced the risk of complications associated with prolonged duration of external fixator. In our cases, percentage and severity of joint stiffness, pin tract infections, pain, soft tissue dystrophy, and disuse osteoporosis were very low. And complications requiring surgical interventions were not observed.

However, one possible drawback of our technique might be its limited role in repairing wounds with soft tissue defect. There were two patients combining soft tissue defects which might well be cured with conventional Ilizarov method; based upon our experience, it turned out that the wounds were only downsized when the docking was achieved with our method. The wounds healed after dressing change for 42 and 28 days respectively. On the bright side, the relatively limited range of soft tissue transport might be beneficial to avoiding the invagination of the soft tissue in the docking site. In our study, only minimal soft tissue invagination was observed between the bone ends in patients with intact soft tissue coverage.

This established surgical technique is seldom adopted in the treatment of chronic osteomyelitis because of the risk of incomplete debridement and sequestrectomy which may result in recurrence of infection. We believe that this risk was overcome with the several tips used in our surgical procedure. In stage Ι, imaging examinations especially PET-CT made a great significance in preoperative preparation to assess the extent of infection and determine the resection levels. It was also very critical to perform radical debridement and removal of all infected and dead tissue in strict accordance with the debridement technique described by Cierny et al. [3]. Before stage II, we determined the infection was completely eradiated through three aspects including wound observation for 3 to 6 weeks, laboratory examinations, and frozen-section and Gram-staining of biopsy specimens.

**Fig. 7:**
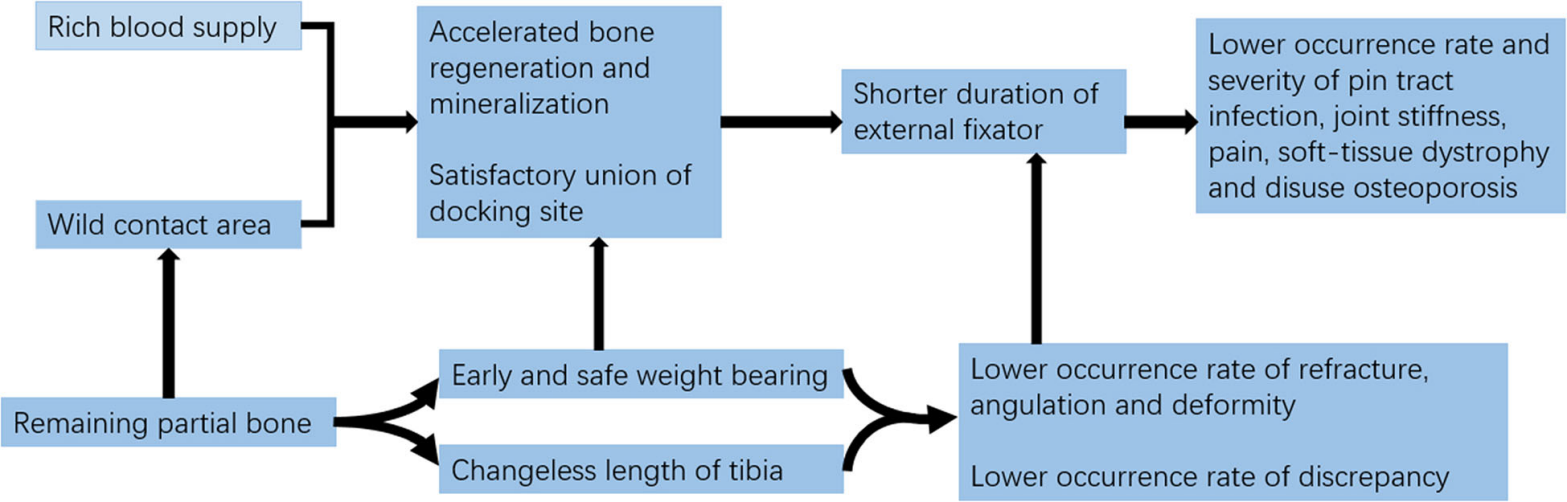
The possible advantages of L-shaped corticotomy with bone flap sliding in the management of chronic tibial osteomyelitis

The technique of vascularized bone flap sliding is proved effective with lots of advantages in our study. It can eliminate dead space, bridge bone defects, promote bone regeneration, protect against infection by ensuring blood supply, allow early rehabilitation and dismantlement of the external fixator, reduce the incidence of complications, and lead to good clinical results (Fig. 7). Though similar technique and excellent results have been reported previously [18], we provided a more detailed description of the technique and made some modification. In conclusion, we represent a feasible alternative method for the treatment of chronic tibial osteomyelitis involving the anterior tibial cortex with intact and healthy posterior cortex.

## Conclusions

The shortcomings of our study are the lack of a direct comparison with a control group and the small number of cases. Also, our operation method may have higher technical requirement than conventional technique of total segment resection. Nevertheless, this surgical technique of vascularized bone flap sliding is a feasible alternative method for the treatment of chronic tibial osteomyelitis involving the anterior tibial cortex with intact and healthy posterior cortex. It allows for earlier removal of the frame and a reduced EFI without recurrence of infection.
